# Reversal of surgical biliary diversion with ileal bile acid transport inhibitors: A new chapter in progressive familiar intrahepatic cholestasis type 1 management?

**DOI:** 10.1002/jpr3.70236

**Published:** 2026-08-31

**Authors:** Teresa Botelho, Giulia Jannone, Roberto Tambucci, Catherine de Magnee, Isabelle Scheers, Etienne Sokal, Xavier Stephenne

**Affiliations:** ^1^ Pediatric Liver Transplant and Hepatology Unit Unidade Local de Saúde de Coimbra Coimbra Portugal; ^2^ Pediatric Gastroenterology and Hepatology, Department of Pediatrics Cliniques Universitaires Saint‐Luc Brussels Belgium; ^3^ Member of the European Reference Network Rare Liver; ^4^ Member of the European Reference Networt TransplantChild; ^5^ Pediatric Surgery and Transplantation Unit, Department of Surgery Cliniques Universitaires Saint‐Luc Brussels Belgium

**Keywords:** genetic liver disorders, liver transplantation, quality of life

## Abstract

Progressive Familial Intrahepatic Cholestasis type 1 (PFIC1) is a multisystem disorder. Although liver transplant (LT) resolves the hepatic disease, post‐LT complications may occur, including severe enteropathy and graft steatosis caused by impaired bile acids handling by the native intestine. Surgical biliary diversion (SBD) is commonly used to alleviate these complications but may result in long‐term morbidity, such as fat‐soluble vitamin deficiency and essential fatty acids malabsorption. We report a PFIC1 patient who developed post‐LT protein‐losing enteropathy, initially managed with SBD. While effective, SBD led to severe fat malabsorption requiring intravenous lipid supplementation. After initiation of ileal bile acid transport inhibitor (IBATi) therapy, SBD was surgically reversed without recurrence of enteropathy. This case highlights IBATi as a potential alternative to SBD and as a strategy to reverse prior surgical interventions and improve quality of life.

## 1 INTRODUCTION

Progressive familial intrahepatic cholestasis (PFIC) comprises a heterogeneous group of autosomal recessive disorders characterized by impaired bile formation/secretion.^1^ PFIC type 1 (PFIC1) is caused by mutations in the ATP8B1 gene encoding the FIC1 protein.^2^ As FIC1 is present in extrahepatic tissues, including the intestine and pancreas,^1^ PFIC1 is a multisystem disorder. While liver transplant (LT) effectively treats the hepatic disease, extrahepatic manifestations may persist or arise posttransplant. Notably, post‐LT enteropathy can induce severe diarrhea and malabsorption, resulting in nutritional deficiencies, failure to thrive, and graft steatosis, which may progress to cirrhosis and graft failure.^2^, ^3^ These complications may result from the inability of the FIC1‐deficient intestine to handle the restored physiological bile acids flow.^2^

Management of post‐LT enteropathy typically relies on nutritional support and attempts to interrupt the enterohepatic bile acids circulation. Surgical biliary diversion (SBD)—including external (EBD) and internal (TIBD) techniques—has been widely used post‐LT in PFIC1 patients. By diverting bile away from the distal ileum, SBD reduces the bile acid load, improves diarrhea, and helps to prevent or treat graft steatofibrosis.^3^, ^4^ However, SBD can also induce fatty acids and fat‐soluble vitamin malabsorption due to a reduced bile flow in the small intestine.

Recently, ileal bile acid transport inhibitors (IBATi) have emerged as a promising therapeutic option.^5^ By inhibiting the apical sodium‐dependent bile acid transporter (ASBT/IBAT) in the terminal ileum, IBATi reduce bile acid reabsorption and interrupt the enterohepatic circulation while preserving intraluminal bile flow, thereby avoiding fatty acid malabsorption.^5^ Recent reports suggest that IBATi may be effective in managing post‐LT complications such as chronic diarrhea in PFIC1 patients.^6^

## 2 CASE REPORT

As reported by Nicastro et al., a male infant with PFIC1 underwent LT at 43 months of age for pruritus refractory to UDCA and rifampicin. Post‐LT, pruritus and hyperbilirubinemia resolved rapidly, but he developed secretory diarrhea attributed to restored bile flow through the native intestine. By 4 years of age, persistent diarrhea led to severe hypoalbuminemia due to protein‐losing enteropathy, and liver biopsy revealed marked macrovesicular steatosis with portal fibrosis. Two years post‐LT, a total EBD was performed, resulting in complete resolution of enteric protein loss within 6 months and marked improvement of steatosis with stable fibrosis on repeat liver biopsy.

Four years post‐LT, EBD was converted to a TIBD to improve quality of life. Although SBD resolved the protein‐losing enteropathy and improved hepatic steatosis, it was complicated by severe fat malabsorption despite supplementation. Five years later, the patient developed photophobia and nyctalopia, resolving after vitamin A administration. In this context, the severe depressive status of the patient was hypothesized to be exacerbated by essential fatty acids deficiency; biweekly intravenous lipid supplementation led to mood stabilization, weight gain, and improved nutritional markers. Nevertheless, intermittent bile acid‐mediated diarrhea and abdominal pain persisted, and long‐term dependence on intravenous lipids significantly impaired the patient's daily life.

**Figure 1:**
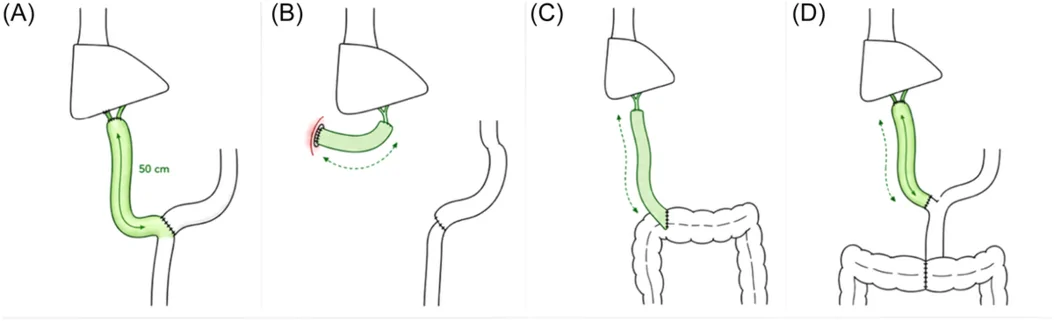
The surgical procedures. (A) Liver transplantation with Roux‐en‐Y hepaticojejunostomy (50 cm transmesocolic limb); (B) Conversion to external biliary diversion by takedown of the hepaticojejunostomy and exteriorization of the Roux‐en‐Y limb as a jejunostomy; (C) Conversion to internal biliary diversion through end‐to‐side jejuno‐colic anastomosis, restoring internal bile drainage; (D) Reversal of the internal biliary diversion with restoration of the Roux‐en‐Y configuration and physiological bile flow into the jejunum.

Fifteen years after LT, IBATi became available, and odevixibat was initiated, with the aim of reversing the SBD and thereby resolving the patient's fat malabsorption, without re‐inducing a post‐LT protein‐losing enteropathy. Treatment was well tolerated, with no gastrointestinal worsening. Two months later, TIBD was surgically reversed, restoring intestinal continuity. Intravenous lipid supplementation was successfully discontinued without recurrence of post‐LT enteropathy‐related malabsorption. The surgical procedures are illustrated in Figure 1.

**Figure 2:**
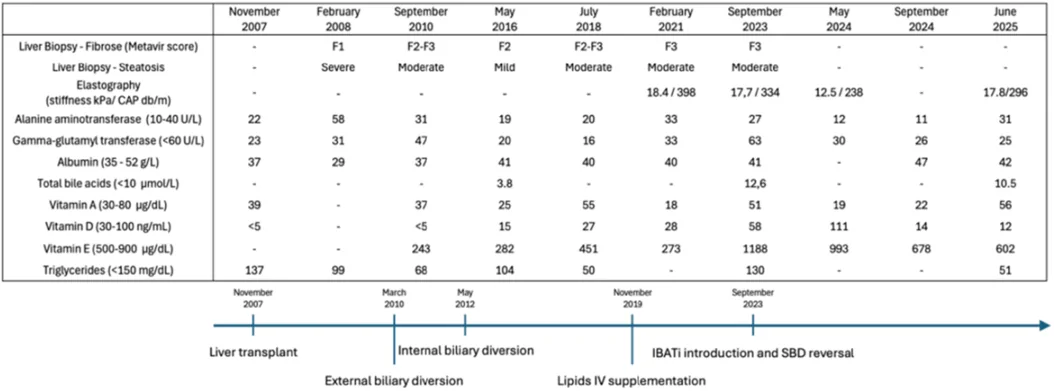
Timeline of therapeutic interventions in relation to histological, elastography, and biological values of the patient. CAP, controlled attenuation parameter.

Twenty months after SBD reversal and IBATi initiation, gastrointestinal and depressive status remain stable. Albumin and liver function tests are normal, and liver elastography remains stable (Figure 2). This case highlights that certain challenging scenarios may necessitate sequential interventions, and supports the feasibility of SBD reversal in PFIC1 patients after LT.

## 3 DISCUSSION

Patients with PFIC1 undergoing LT remain particularly challenging due to the persistence or development of extrahepatic morbidities, notably chronic diarrhea, malabsorption, and graft steatosis, which markedly impair quality of life and long‐term outcomes.

In clinical practice, SBD has been performed to interrupt the enterohepatic circulation and improve cholestasis‐related symptoms (pre‐LT)^5^ or bile acid‐mediated enteropathy (post‐LT).^2^ It proved effective in controlling severe diarrhea and reversing graft steatosis in PFIC1 patients after LT.^2^, ^7^ However, SBD is associated with surgical risks and potential long‐term complications, including electrolyte imbalances, dehydration, high‐output bile losses, malnutrition and fat‐soluble vitamin deficiencies, cholangitis, prolapse or stoma bleeding (with EBD), and bile acid‐driven diarrhea (with TIBD).^8^, ^9^ By diverting bile away from the small intestine, SBD can impair fat absorption and create a need for long‐term IV supplementation of essential fatty acids and liposoluble vitamins, representing a major burden for patients and families.

IBATi therapy offers a pharmacological option to interrupt the enterohepatic circulation, partially mimicking the effects of SBD.^5^ Clinical trials have demonstrated IBATi efficacy in improving pruritus in cholestatic disorders,^8^ and recent data support their role in managing post‐LT PFIC1 complications including reduced diarrhea and improved graft steatosis.^10^ Ohlendorf et al reported successful reduction of post‐LT diarrhea and growth failure with IBATi in a PFIC1 patient.^6^ Our case extends these observations by showing that IBATi can not only manage symptoms typically addressed by SBD, but also enable reversal of a preexisting biliary diversion without recurrence of enteropathy.

While current evidence supports IBATi as a potential alternative to SBD,^8^ our case goes one step further and suggests the possibility of transitioning from SBD to IBATi. To our knowledge, this is the first case demonstrating successful SBD reversal under IBATi therapy, allowing the resolution of SBD‐related side effects without enteropathy recurrence. This case report has limitations inherent to its single‐patient design, and further studies are required to assess long‐term outcomes following SBD reversal in PFIC1 patients treated with IBATi after LT.

## 4 CONCLUSIONS

Severe diarrhea and malabsorption are major complications following LT in PFIC1 and often require SBD, which may itself lead to significant side effects. This case demonstrates that initiation of IBATi therapy followed by reversal of a preexisting TIBD can successfully resolve both SBD‐related complications and post‐LT enteropathy. These findings support a novel therapeutic approach in which IBATi may serve not only as an alternative to SBD but also as a means to safely discontinue prior biliary diversion in selected PFIC1 patients after LT.

## CONFLICT OF INTEREST STATEMENT

The authors declare no conflicts of interest.

## ETHICS STATEMENT

Written informed consent was obtained from the patient.
